# Systematic Reviewers in Clinical Neurology Do Not Routinely Search Clinical Trials Registries

**DOI:** 10.1371/journal.pone.0134596

**Published:** 2015-07-30

**Authors:** Philip Marcus Sinnett, Branden Carr, Gregory Cook, Halie Mucklerath, Laura Varney, Matt Weiher, Vadim Yerokhin, Matt Vassar

**Affiliations:** Oklahoma State University Center for Health Sciences, Tulsa, OK, United States of America; Universitat Wien, AUSTRIA

## Abstract

We examined the use of clinical trials registries in published systematic reviews and meta-analyses from clinical neurology. A review of publications between January 1, 2008 and December 31, 2014 from five neuroscience journals (*Annals of Neurology*, *Brain*, *Lancet Neurology*, *Neurology*, and *The Neuroscientist*) was performed to identify eligible systematic reviews. The systematic reviews comprising the final sample were independently appraised to determine if clinical trials registries had been included as part of the search process. Studies acknowledging the use of a trials registry were further examined to determine whether trial data had been incorporated into the analysis. The initial search yielded 194 studies, of which 78 systematic reviews met the selection criteria. Of those, five acknowledged the use of a specific clinical trials registry: four reviewed unpublished trial data and two incorporated unpublished trial data into their results. Based on our sample of systematic reviews, there was no increase in the use of trials registries in systematic review searches over time. Few systematic reviews published in clinical neurology journals included data from relevant clinical trials registries.

## Introduction

Researchers attempt to locate all relevant data to incorporate into a systematic review or meta-analysis. This comprehensive approach to searching is intended to provide a more balanced and representative estimate of the true effect of an intervention. A common methodological feature of data synthesis is an over-reliance on published study results in determining an aggregate summary effect. There is, however, a trend toward publishing statistically significant outcomes more often than those that support the null hypothesis, an issue known as publication bias and defined by Dickersin as “the tendency on the parts of investigators, reviewers, and editors to submit or accept manuscripts for publication based on the direction or strength of the study findings” [1]. Thus, the summary effect may be misrepresented if only published studies yielding statistically significant outcomes are included.

While there have been several proposed strategies for identifying additional information sources to address publication bias, little attention has been given to clinical trials registries as a data source. In 2005, the International Committee of Medical Journal Editors (ICMJE) required that all primary, prospective clinical trials involving human participants register their protocols prior to conducting the study as a necessary condition for publication among participating journals [2]. Furthermore, federal mandates in the United States require that clinical trials now be registered via passage of the 2007 Food and Drug Administration Amendments Act (FDAAA) [3]. Most recently, the World Health Organization released a position statement calling for registration of all clinical trials in a publically available, free to access, searchable clinical trials registry [4]. Such advancements make clinical trials registries a worthwhile source of unpublished trial data for consideration in evidence synthesis research.

Clinical neurology is a specialty of medicine where less is known about publication bias. In 2006, Liebeskind, Kidwell, Sayre, and Saver [5] examined clinical trials of acute ischemic stroke for evidence of publication bias. Based on 178 trials across 75 agents and non-pharmacologic interventions, the authors found evidence for publication bias due to an underreporting of smaller, non-beneficial studies. Publication bias has also been noted in preclinical studies of stroke [6]. O’Collins et al. [7] conducted a review of 1,026 experimental treatments in translational stroke research and found that between 62% and 74% of preclinical models found positive results in favor of the experimental condition. Given limited evidence concerning publication bias in the neurology literature, we examined the use of clinical trials registries in systematic review searches in the clinical neurology literature from 2008 to 2014 and investigated whether eligible trials were being found within these registries. As preclinical trials registries are still in their infancy and systematic review methodology is only beginning to make its way to the preclinical domain, we could not examine these issues here.

## Methods

We identified systematic reviews and meta-analyses published between January 1, 2008 and December 31, 2014, in the following publications: *Annals of Neurology*, *Brain*, *Lancet Neurology*, *Neurology*, and *The Neuroscientist*. We conducted a MEDLINE database search on PubMed using the following search string: meta-analysis[Title/Abstract]) OR meta-analysis[Publication Type]) OR systematic review[Title/Abstract])) AND ("Neurology"[Journal]) OR ("The Neuroscientist: a review journal bringing neurobiology, neurology, and psychiatry"[Journal])) OR "The Lancet. Neurology"[Journal]) OR "Annals of neurology" [Journal]) OR "Brain: a journal of neurology" [Journal]) and imposing a date limiter as mentioned above. This search strategy was adapted from a published search protocol that has been validated as sensitive to identifying published meta-analyses and systematic reviews [8]. This search was performed on Monday, December 29, 2014.

### Training

Prior to commencement of the study, we held a training session for all coders to improve the consistency and accuracy in the screening and abstraction processes. An abstraction manual was developed to standardize coding practices and ensure that coders adhered to predefined data entry standards. A subset of eight articles was selected for training purposes and used during the training session to familiarize coders with the process.

### Screening

**Fig 1:**
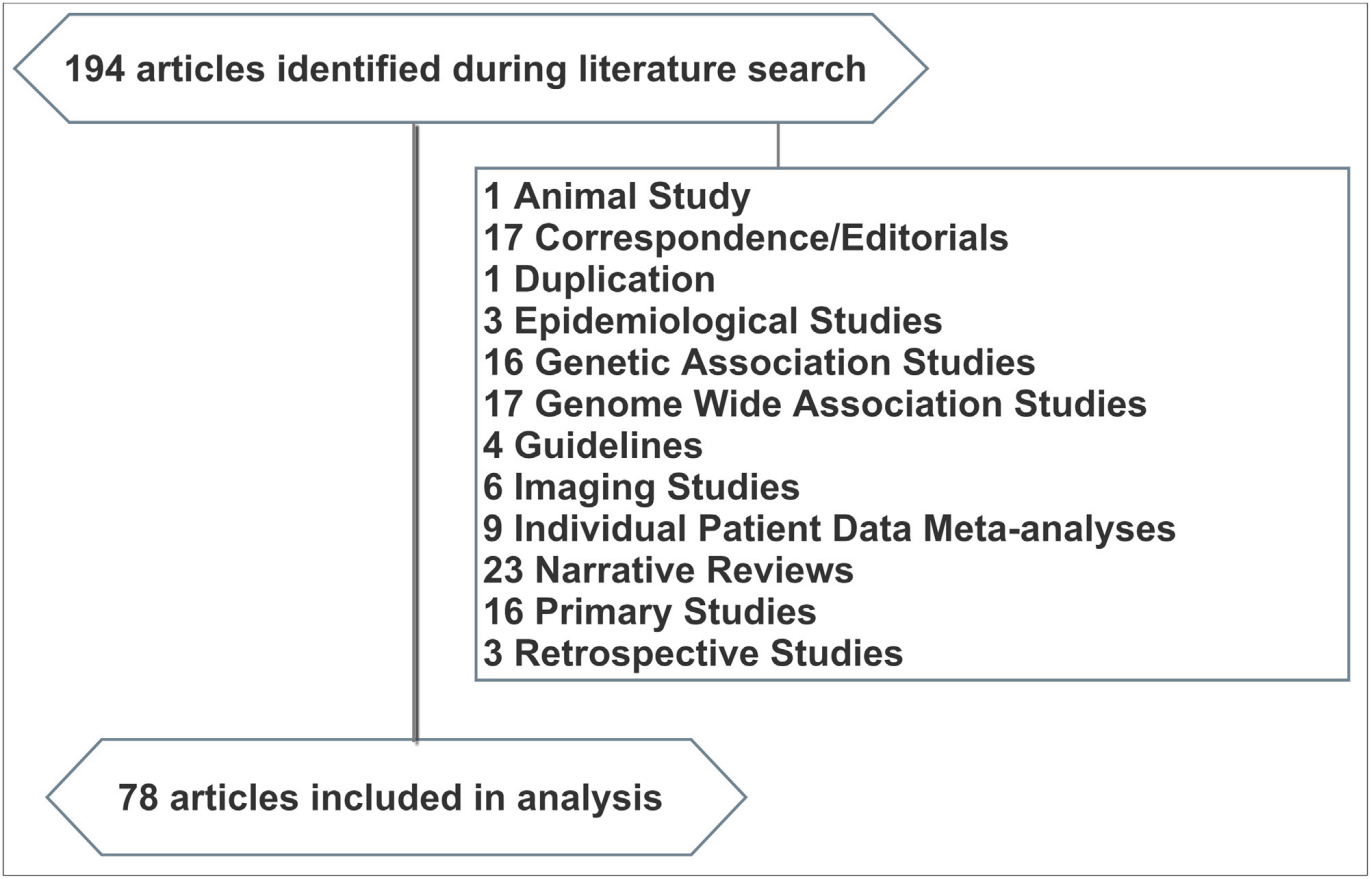
Flow diagram of Study Eligibility.

Following the training exercise, each article (N = 194) was screened to determine whether it met inclusion criteria as a meta-analysis or systematic review. Specific study types were excluded from this review since such studies were either not research syntheses or otherwise unlikely to search clinical trials registries for unpublished data (Fig 1). The final sample consisted of 78 meta-analyses and systematic reviews.

### Coding

After screening, each coder first independently coded a subset of the total articles based on the standardized approach of the abstraction manual used in training. These coded elements included the authors’ names, journal name, article title, year of publication, whether a clinical trials registry search was conducted, and, if so, the specific name of the registry. Next, a validity check was performed, and each coded element was individually verified by a second reviewer. Finally, a comprehensive review of all studies was conducted jointly by the two principal investigators to ensure that all studies met inclusion criteria and that all coded elements were correctly entered. This stepwise process was used to ensure the accuracy of the coded data.

Following the work of Jones et al. [9], we used the World Health Organization’s Trial Registries List Version 2.1 [10] to determine eligible registries for our study. These registries met specific criteria for quality and validity, content, accessibility, unambiguous identification, technical capacity, and administration/governance [11]. Clinicaltrials.gov was also considered an eligible registry. Though sometimes referenced in studies included in our review, sources such as the Cochrane CENTRAL Registry of Controlled Trials were not included as clinical trials registries, but treated as a bibliographic database, as CENTRAL features a selection of already published trials. Studies that reported non-specific use of clinical trials registries were not included in our evaluation. Data from this study have been made publically available on Figshare (http://dx.doi.org/10.6084/m9.figshare.1476896).

## Results

**Fig 2:**
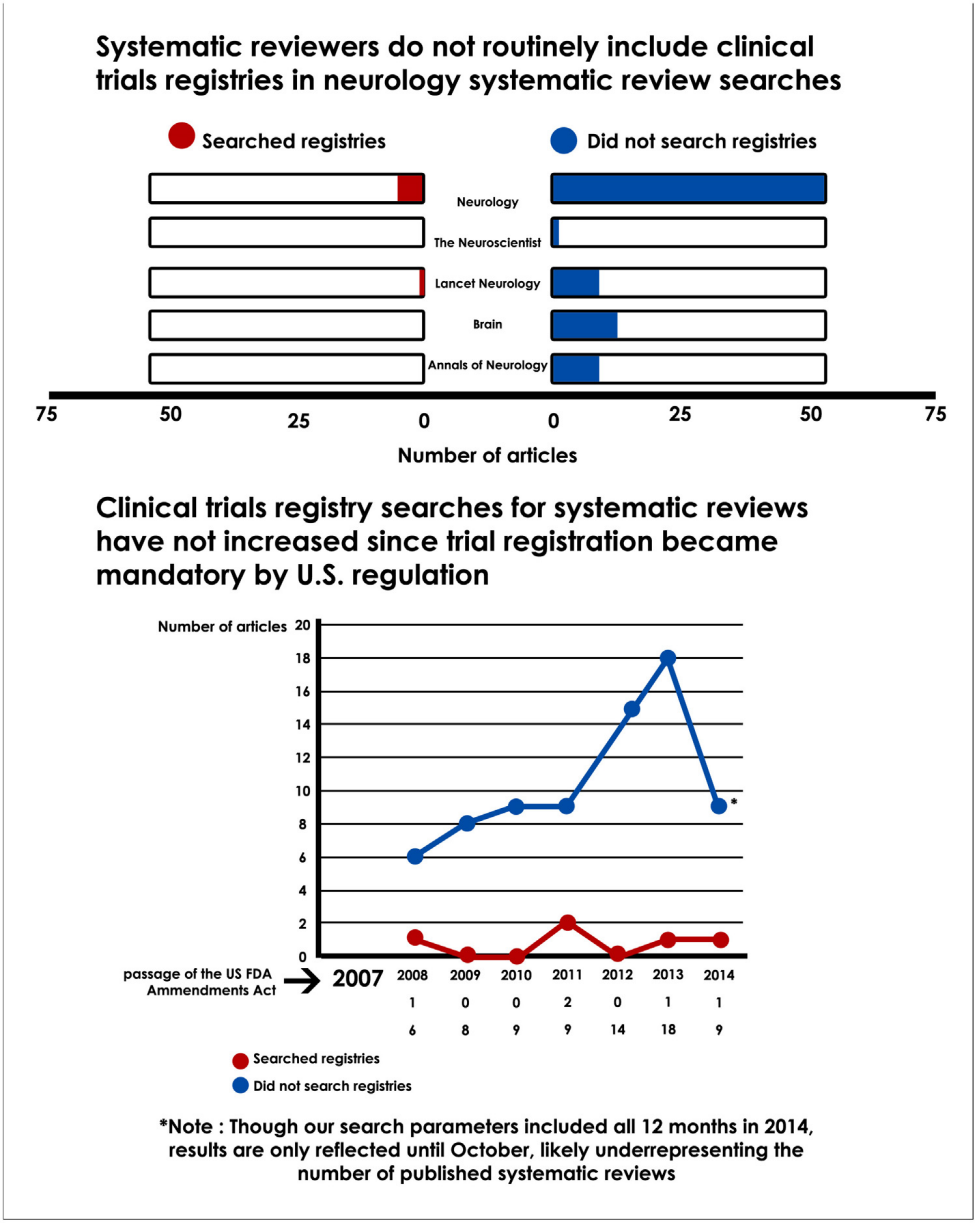
Clinical Trials Registry Use in Neurology Systematic Review Searches.

We first investigated the frequency of use of clinical trials registries as part of the systematic review search process. Fig 2 graphically displays these results. Of the studies included in our analysis, only five acknowledged use of a specific clinical trials registry. These studies searched two of the clinical trials registries discussed previously. Clinicaltrials.gov was used most frequently (cited in four studies), followed by ISRCTN (cited in two studies). When examining the use of trials registries by journal, four systematic reviews of fifty-seven found in the journal *Neurology* reported the use of a clinical trials registry. All four referenced clinicaltrials.gov, and one study also reported a search of the ISRCTN registry. Of the ten studies retrieved from *Lancet Neurology*, one reported the use of both ISRCTN and clinicaltrials.gov in their report. Of the remaining eleven studies—nine retrieved from *Annals of Neurology*, one identified in *Brain*, and one found in *The Neurologist*—none reported use of a specific clinical trials registry.

Next, we investigated whether those systematic reviews that searched trials registries located and incorporated data from these registries into their results. Four out of five located potentially relevant trials from a clinical trials registry. Two of these included unpublished trial data in their final analysis. The first identified four unpublished studies—three from clinicaltrials.gov and one from another publication—and used one trial from clinicaltrials.gov in the final analysis. The second identified two ongoing clinical trials that were only mentioned as discussion. Of the three studies that did not include unpublished data, one identified seventeen unpublished studies from trials registries as reported in a flow diagram. None were included in their analysis. The next identified and excluded three in-progress trials from an unknown source. The last was unclear whether they identified trials from registries.

Finally, we wanted to investigate the use of clinical trials registries by year, focusing on the period between from 2008 and 2014. The year 2007 is significant for the passing of Federal mandate FDAAA 801, which requires the registration of all clinical trials in the United States with the Federal government. Thus, we examined clinical trials registry use starting the year following the legislation to investigate whether the frequency of use of clinical trials registries in systematic review searches had increased during that time. Fig 2 shows a graphical representation of trends in clinical trials registry use since 2008. Notably, it can be seen that while the publication of systematic reviews and meta-analyses have increased over time, the frequency of use of clinical trials registries within these types of studies has not.

## Conclusion

Our results indicate that clinical trials registries are underused in systematic reviews and meta-analyses within neuroscience journals, although this result should be interpreted within the context of our sample. As such, there is risk of publication bias due to the possible omission of negligible, unpublished, or non-significant results. This has the potential to skew data toward more statistically significant results, allowing for misrepresentation of the aggregate summary effect. This, in turn, poses concern regarding the integrity of the results and impairs the ability to draw clinically relevant conclusions from the study. The use of clinical trials registries in systematic reviews in neurology is, therefore, a valuable, though consistently overlooked, method to accumulate data. Interestingly, of reviewed studies that searched at least one trials registry, most located trials that met at least initial eligibility criteria and two studies incorporated these trials into their systematic review. This finding suggests that evidence synthesis researchers who use trials registries are finding potentially relevant data for inclusion in their research. Finally, we did not detect any trend in the use of clinical trials registries over time since the passage of the FDA mandate. With 187,856 studies currently listed with clinicaltrials.gov [12], it seems as though valuable data are currently omitted from consideration by evidence synthesis researchers.

Further investigation is needed to assess the extent of publication bias within neurology journals. Additionally, reanalyzing meta-analytic results while incorporating data from registered, but unpublished clinical trials could yield interesting comparisons between original and revised summary effects. For example, Hart, Lundh, and Bero [13] reanalyzed 42 meta-analyses of drug trials after incorporating unpublished FDA trial data. Nineteen meta-analyses showed lower efficacy of the drug, three showed identical efficacy, and nineteen showed greater efficacy. It would be beneficial to perform a similar investigation of clinical neurology meta-analyses to examine changes in outcomes.

Additionally, future research should examine factors that may limit the use of trials registries by systematic reviewers and information specialists. For example, it is possible that limited search capabilities of trials registries might impede their use. It is also feasible that locating data through trials registries would require contacting the principal investigators to obtain trial data, which could be problematic. Schroll, Bero, and Gøtzsche [14] conducted a survey of Cochrane reviewers to examine the experiences of retrieving and using unpublished trial data in systematic reviews. They found that the majority of reviewers reported searching for unpublished data, primarily by contacting trialists (73.9%); however, non-commercial trial registers accounted for only 6.3% of cases. The authors concluded that searching a trial register is “a good idea and is not time-consuming” (p. 4).

In summary, we found that clinical trials registries were underused in our sample of systematic reviews. Of the few studies that searched trials registries, most located potentially relevant unpublished trial data and, in some cases, incorporated these data into their analyses. We recommend the use of trials registries in systematic review searches (alongside [14]) to help mitigate publication bias in future reviews.
